# The characteristics and the multiple functions of integrin β1 in human cancers

**DOI:** 10.1186/s12967-023-04696-1

**Published:** 2023-11-06

**Authors:** Li Sun, Shuwei Guo, Yiping Xie, Yongliang Yao

**Affiliations:** 1 Department of Clinical Laboratory, Kunshan First People’s Hospital, Affiliated to Jiangsu University, Kunshan, 215300 People’s Republic of China; 2 Department of Clinical Laboratory, Jiangsu Province Hospital of Chinese Medicine, Nanjing, 210029 People’s Republic of China

**Keywords:** Tumor microenvironment, Integrin β1, Extracellular matrix, Clinical significance, Drug resistance

## Abstract

Integrins, which consist of two non-covalently linked α and β subunits, play a crucial role in cell–cell adhesion and cell-extracellular matrix (ECM) interactions. Among them, integrin β1 is the most common subunit and has emerged as a key mediator in cancer, influencing various aspects of cancer progression, including cell motility, adhesion, migration, proliferation, differentiation and chemotherapy resistance. However, given the complexity and sometimes contradictory characteristics, targeting integrin β1 for therapeutics has been a challenge. The emerging understanding of the mechanisms regulating by integrin β1 may guide the development of new strategies for anti-cancer therapy. In this review, we summarize the multiple functions of integrin β1 and signaling pathways which underlie the involvement of integrin β1 in several malignant cancers. Our review suggests the possibility of using integrin β1 as a therapeutic target and highlights the need for patient stratification based on expression of different integrin receptors in future clinical studies.

## Background

Integrins, comprised of α and β subunits non-covalently bound together, form heterodimeric complexes found in endothelial cells, pericytes, fibroblasts, and tumor cells. In mammals, there are a total of 18 α subunits and 8 β subunits. Through their mutual combinations, at least 24 αβ integrin heterodimers are formed. Of these, half contain the β1 subunit [1]. The β subunit consists of a plexin-semaphorin-integrin domain, a hybrid domain, an I-like domain which is inserted in the hybrid domain and is homologous to the αI-domain of the α subunit, and also EGF1-4 and β tail domains. The α subunit is composed of an extracellular domain consisting of a seven-bladed β-propeller head domain, a thigh domain and two calf domains (calf 1 and calf 2). The αI domain, containing approximately 200 amino acids, is inserted between β propeller blades 2 and 3. The αI-domain contains a metal ion-dependent adhesion site, which participates in ligand binding [2]. Both α and β subunits have large extracellular domains, enabling them to sense and respond to stimuli from extracellular matrix (ECM) components such as collagen, fibronectin, fibrinogen, laminin and vitronectin. Furthermore, research has revealed that integrins contain a transmembrane domain and a short cytoplasmic domain which play a central role in signal transduction involving FAK, AKT, MAPK, and Src family kinases, thus regulating cell survival, migration, immune escape, and resistance to radiotherapy and chemotherapy [3].

**Fig. 1:**
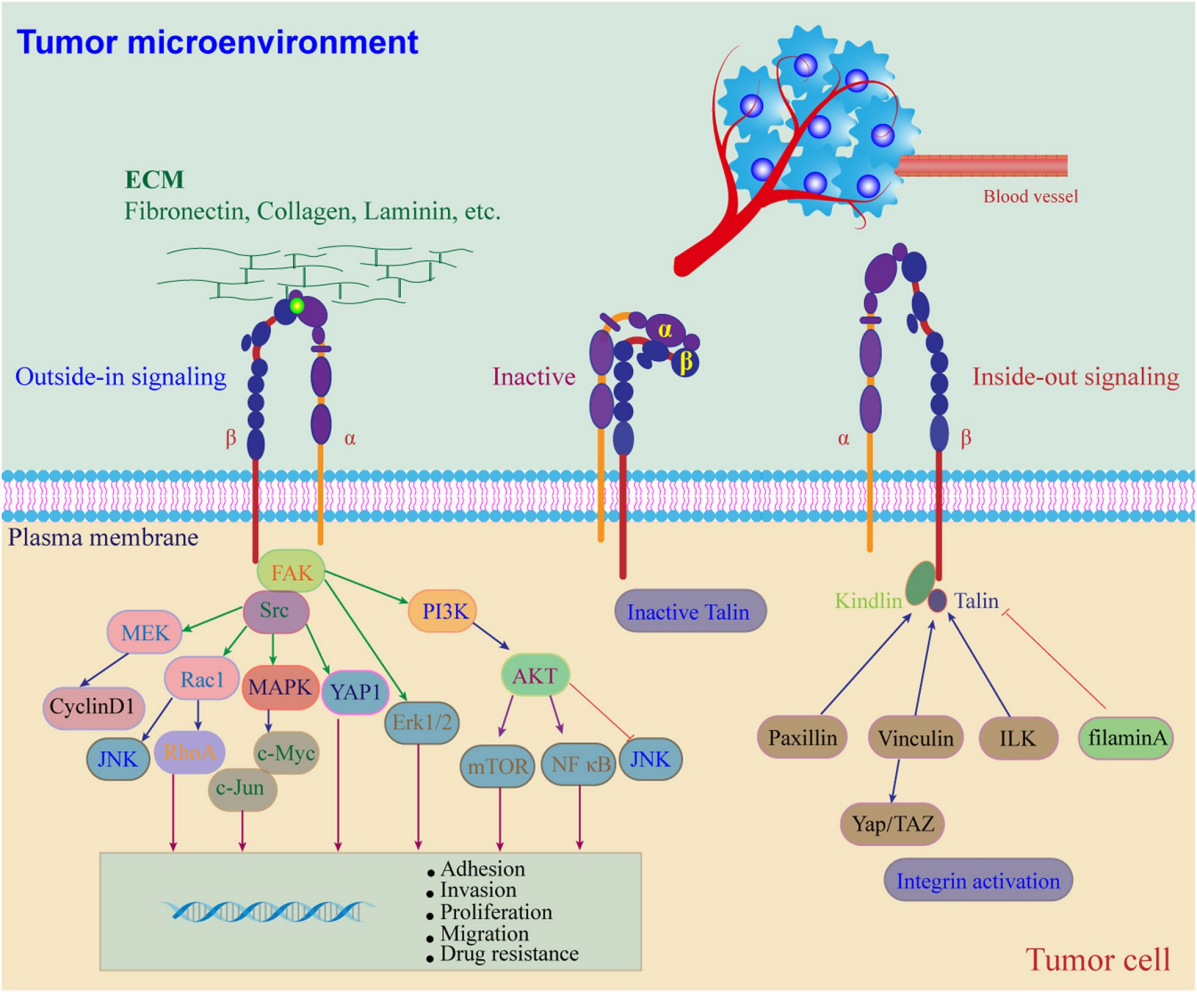
Framework diagram of cellular signaling triggered by integrin β1 in tumor microenvironment. During the cell adherence to ECM, integrin β1 activity undergoes conformational changes that induce cellular signaling including “inside-out” signal in which signals from inside the cell activate the integrin for binding to the extracellular ligands and “outside-in” signal in which extracellular ligand interaction activates integrin receptor by separating the integrin cytoplasmic domain triggering the intracellular signaling molecules

The expression and function of the major integrins and their relationship to tumor types and metastatic sites are different. For example, the progression of liver and endometrial cancer is mainly related to integrin αvβ6, while thyroid cancer is associated with integrin α6β4. Integrin αvβ3 plays a vital role in cervical cancer and bone metastasis of tumors, as does integrin αvβ6 [4]. In addition, integrin β1, also recognized as CD29, which is one of the most common subunits in the integrin family and is composed of a β1 subunit and different α subunits, plays a non-negligible role in crucial developmental pathways. Integrin β1 is a human protein-coding gene with a total length of 58048 bp, located on human chromosome 10p11.2 and consisting of 18 exons. Moreover, its mRNA encodes approximately 798 amino acids, with a molecular weight ranging from 100 to 132 kDa [5]. This gene has three transcript variants, including transcript variants 1A, 1E, and 1D. Transcript variant 1A has a full length of 3735 bp, contains 16 exons, and encodes a protein of 798 amino acids; transcript variant 1E has a full length of 3794 bp and encodes a protein of 798 amino acids; transcript variant 1D has a full length of 3739 bp and encodes a protein of 801 amino acids [6]. The primary function of integrin β1 is to facilitate adhesion between cancer cells and the ECM, forming the basis for cancer cell survival. It is closely associated with cancer cell metastasis, radiotherapy, chemotherapy, and targeted therapy, among other activities [7]. When cancer cells adhere to the ECM, two types of cellular signaling are triggered by integrin β1 activity: an “inside-out” signal in which signals from inside the cell activate the integrin for binding to extracellular ligands, and an “outside-in” signal in which the extracellular ligand interacts with the integrin receptor, causing the integrin cytoplasmic domain to separate and thereby activate the integrin receptor and trigger intracellular signaling molecules (Fig. 1). Herein, we provide a systematic and complete review of integrin β1-mediated signal transduction and its role in tumor drug resistance, and highlight ongoing efforts to develop new therapies from bench to clinic.

## Function of the integrin β1 family

Each heterodimer of integrin β1 binds to a specific molecule and follows a unique signaling pathway activation pattern (Table 1). Based on their affinity for ligands, integrins can be categorized into four groups, each with distinct receptors; namely, arginine-glycine-aspartate (RGD)-binding receptors (αvβ1, αvβ3, αvβ5, αvβ6, αvβ8, α5β1, α8β1, and αIIbβ3), leukocyte-specific receptors (the β2 subfamily plus α4β1, α9β1, α4β7, and αEβ7), laminin-binding receptors (α3β1, α7β1, α6β1, and α6β4), as well as collagen-binding receptors (α1β1, α2β1, α10β1, and α11β1) [5, 23]. The expression and functions of integrin β1 in various cancer types were summarized in Table 2. Among these, the intriguing roles of integrin β1, in combination with distinct α subunits, are clarified as follows.

**Table 1:** Signaling pathways mediated by integrin β1 with distinct α subunits

Receptor type	Receptor	Ligand	Signaling pathway	Function	References
RGD receptors	α5β1	N/A	FAK – p53	Promote cancer growth and metastasis	[8]
	α8β1	N/A	CXCR4	Enhance cancer cells migration and invasion	[9]
	αvβ1	Tenascin-C	TGF-β – SMAD2/3	Contribute to the stiffer stromal formation	[10]
Leukocyte-specific receptors	α4β1	VCAM-1	AKT/MAPK/NF-κB	Decrease cancer cells apoptosis	[11]
	α9β1	N/A	FAK – Src-Rac1 – RhoA	Suppress cancer cells migration and invasiveness	[12]
		N/A	ILK – PKA – GSK3	Promote cancer growth and metastasis	[13]
Laminin-binding receptors	α6β1	Laminin	PI3K/NF-κB	Drive cancer cells survival	[14]
	α3β1	CD151	FAK/src – STAT3/AKT	Promote carcinogenesis	[15]
	α7β1	N/A	FAK – MAPK – ERK	Enhance stem cell properties	[16]
Collagen-binding receptors	α10β1	HU177 cryptic collagen epitope	FGF-2 – ERK	Promote tumor growth	[17]
		Collagen II	TRIO – RAC –RICTOR – mTOR	Promote cancer cells survival	[18]
	α1β1	Collagen V	ERK1/2	Enhance cancer cells invasion	[19]
		Collagen I	FAK/Src and p130Cas/JNK	Enhance cancer cells invasion	[20]
	α2β1	Collagen I	JAK – STAT3	Strengthen cancer cells proliferation and tumorigenesis	[21]
	α11β1	N/A	Src – YAP1	Promote tumor growth	[22]

*N/A* not available

**Table 2:** The expression and functions of integrin β1 in various cancer types

Tumor type	Expression	Ligand/receptor	Receptor type	Function	Reference
Pancreatic cancer	up	Muc5ac – CD44/ integrin β1	N/A	Promote cancer progression and chemoresistance	[24]
Acute myeloid leukaemia	up	Fibronectin – integrin β1	N/A	Confer radiation and chemoresistance	[25]
Hepatocellular cancer	up	Integrin β1	N/A	Accelerate tumor growth	[26]
Ovarian cancer	up	VCAM-1 – integrin α4β1	Leukocyte-specific receptors	Cause chemotherapy resistance and metastasis	[27]
Breast cancer	up	Collagen I – integrin β1	Collagen-binding receptors	Drive invasion, metastasis, angiogenesis, and drug resistance	[28]
Lung cancer	up	Integrin α9β1	Leukocyte-specific receptors	Promote tumor growth and metastasis	[29]
	down	Integrin α8β1	RGD receptors	Negatively related to tumor progression	[30]
Colon cancer	up	Integrin α2β1	Collagen-binding receptors	Promote tumor growth and liver metastasis	[31]
Glioblastoma	up	Hsc70 – integrin α5β1	RGD receptors	Enhance invasion	[32]

*N/A* not available

### Integrins α5β1, α8β1, and αvβ1 in RGD receptors

#### The role of integrins α5β1, α8β1, and αvβ1 in tumor progression

RGD receptors which recognize the triplet sequence RGD motif, are found in many ECM proteins such as fibronectin, collagen, vitronectin, osteopontin and thrombospondin [33]. The RGD-binding subfamily members play an important role in angiogenesis and thrombosis and are considered the most essential integrin targets in drug discovery [34]. Currently, anti-integrin drugs designed to block the interaction between integrins and ECM have been developed for the prevention and treatment of various diseases [35, 36]. Moreover, integrins binding to RGD receptors regulate cell proliferation and survival signals, as well as the localization and activation of transforming growth factor-β (TGF-β), supporting angiogenesis [37, 38]. The expression level of integrin α5β1 is higher in liver cancer tissues than in paired adjacent tissues, and the interactions between integrin α5β1 and fibronectin promotes tumor growth and angiogenesis [39]. Immunohistochemistry analyses have confirmed that integrin α5β1 is overexpressed in esophageal squamous cell cancer, with high expression being linked to a poor prognosis and potentially serving as an independent prognostic factor [40]. Immunoprecipitation and mass spectrometry have revealed that all monoclonal antibodies recognized integrin α5β1 and blocking α5 in diffuse-type gastric cancer cells or fibronectin deposited on cancer-associated fibroblasts abrogate the heterocellular interaction [41]. In lung cancer, the expression of α8 subunit is downregulated, and patients with high expression exhibit a favorable prognosis, which is closely linked with the immune microenvironment, tumor heterogeneity, and cancer cell stemness [30]. Simultaneously, the low expression of α8 subunit is correlated with poor disease-free survival in renal cell carcinoma patients [31]. Reports indicate that the overexpression of the α8 subunit induces endothelial-mesenchymal transition (EMT) and enhances cell migration and invasion in early relapsed multiple myeloma patients [9]. Accordingly, the expression of the α8 subunit is closely linked to the occurrence of colorectal cancer [42]. Integrin αvβ1 is enriched in extracellular vesicles of metastatic breast cancer cells mediated by galectin-3, and integrin αvβ1 is important for extracellular vesicle retention in ECM [43, 44].

#### Signaling pathways mediated by integrins α5β1, α8β1, and αvβ1

It has been reported that chenodeoxycholic acid attenuate lung cancer pathogenesis via the integrin α5β1/FAK/p53 axis [8]. Ryu et al. indicated that the α8 subunit may regulate CXCR4/SDF-1α signaling, causing multiple myeloma cells to migrate, and also found the crosstalk between the α8 subunit and PDGF receptor may mediate multiple myeloma pathogenesis [9]. Increased levels of integrin αvβ1 heterodimers induced by tenascin-C activated the TGF-β signaling cascade, resulting in the transformation of highly contractile myofibroblasts in breast cancer [10].

### Integrins α4β1 and α9β1 in leukocyte-specific receptors

#### The role of integrins α4β1 and α9β1in tumor progression

Leukocyte-specific receptors are crucial for host defense. Their most prevalent function is to facilitate the recruitment of neutrophils to inflamed tissues and promote phagocytosis of pathogens. Recent data likewise indicate that they play a role in regulating neutrophil apoptosis. Neutrophils are terminally differentiated cells that undergo constitutive apoptosis, and their apoptosis and clearance are essential for inflammation resolution [45]. For example, integrin α4β1, also recognized as very late antigen-4, is a heterodimeric cell surface receptor expressed on most white blood cells, forming the foundation for leukocyte homing, migration, differentiation, activation, and survival [46]. In bone marrow samples from patients with primary acute myeloid leukemia, CD44 engagement by hyaluronan is involved in inducing the inside-out activation of integrin α4β1, thereby enhancing leukemia cell adhesion to vascular cell adhesion molecule-1 (VCAM-1) [11]. Integrin α4β1 is also a major adhesion receptor mediating multiple myeloma cell-stromal interactions, and its expression and function are downregulated by bortezomib, an anti-multiple myeloma agent, leading to inhibition of cell adhesion-mediated drug resistance and cell apoptosis [47]. In addition, integrin α4β1 plays a significant role in controlling the positioning of both healthy and malignant B cells within tissues, thereby determining the pattern of organ infiltration [48]. In chronic lymphocytic leukemia, the level of integrin α4β1 was determined by measuring the expression of the CD49d chain by flow cytometry. The results illustrated that higher levels of integrin α4β1 were associated with a worse prognosis, consistent with its crucial role as a key molecule facilitating protective niche formation of lymphocytic leukemia cells in the bone marrow and lymph nodes [49]. The α9 subunit used to be known as ITGA4L (integrin-α4-like), because the α9 and α4 subunits show peptide sequence similarities and share several common ligands. However, the α9 and α4 subunits exert distinct as well as similar physiological functions [50]. It has been demonstrated that integrin α9β1 functioned as an active heterodimer on the plasma membrane of endometrial stromal, endometrial epithelial, and porcine spermatogonial stem cells in an undifferentiated state [51, 52]. Varney et al*.* reported the critical importance of integrin α9β1 loss in epidermal tumor cells for maintaining persistent stromal vessel density [53]. Additionally, fully activated integrin α9β1 has been correlated with less migratory behavior in melanoma cells [54]. Moreover, there has been a suggestion of a potential role for integrin α9β1 expressed in neutrophils in cases of aspiration pneumonia [55, 56]. Results indicated that integrin α9β1, when in a high activation state, can induce and localize to focal adhesions, but in its intermediate activity state, it typically supports melanoma cell adhesion consistent with migration [57]. Functional studies strongly support the role of integrin α9β1 in the adhesion and differentiation of hematopoietic stem and progenitor cells in the endosteal stem cell niche [58]. Furthermore, it has been proposed that α9 subunit may function as a tumor suppressor gene in nasopharyngeal cancer, influencing tumor cell biology [59]. In various reports, integrin α9β1 has been shown to enhance malignant tumor growth and metastasis, with its expression being increased in highly metastatic triple-negative breast cancer cells [13].

#### Signaling pathways mediated by integrins α4β1 and α9β1

The interaction between integrin α4β1 and VCAM-1 promotes the activation of AKT, MAPK, NF-κB, and mTOR signals, leading to reduced apoptosis in acute myeloid leukemia cells [35]. Moreover, the α9 subunit was observed to suppress hepatoma cell migration and invasiveness through FAK/Src-Rac1/RhoA signaling [12]. α9 subunit depletion, on the other hand, was determined to suppress triple-negative breast cancer growth and metastasis by promoting β-catenin degradation through the ILK/PKA/GSK3 pathway [13].

### Integrins α6β1, α3β1, and α7β1 in laminin-binding receptors

#### The role of integrins α6β1, α3β1, and α7β1 in tumor progression

Laminins are one of major components of the ECM, consisting of glycoproteins with relatively high molecular weights (400–900 kDa) that are typically found in the basement membranes of various epithelial tissues and take the form of a cross or T made up of three interlaced chains (α, β, and γ) [60, 61]. Integrin α6β1 expression in cancer cells has been reported, and it has been argued that it facilitates tumor invasion, angiogenesis, and cancer progression [62]. Laminin-511 and laminin-521 preserve the pluripotency of pluripotent stem cells and human embryonic stem cells via the integrin α6β1/αvβ1 pathways [63]. Integrin α6β1 is highly expressed in metastatic and androgen receptor-positive prostate cancer [14]. Accordingly, integrin α3β1 promotes angiogenesis of glioblastoma-associated endothelial cells through calcium-mediated exocytosis of macropinosomes and lysosomes [64]. Numerous studies have demonstrated that integrin α3β1 supported the motility and invasion of thyroid papillary cancer cells and was involved in tumor progression [65]. Moreover, integrin α3β1 is implicated in regulating tumor-derived proteases bone morphogenetic protein 1, matrix metalloproteinase-9, and matrix metalloproteinase-3 in the secretome of epidermal tumors, making it a potential therapeutic target [66]. Additionally, integrin α3β1 on keratinocytes facilitates the secretion of IL-1α and exerts paracrine regulation of fibroblast gene expression and differentiation [67]. Integrin α3β1 has also been found to induce the Brn-2 transcription factor, thereby promoting invasion and metastatic properties in breast cancer cells [68, 69]. Aberrantly glycosylated integrin α3β1 is a unique urinary biomarker for the diagnosis of bladder cancer [70]. Polymersomal docetaxel targeting integrin α3β1 has emerged as an advanced nanotherapeutic for non-small cell cancer treatment [71]. Meanwhile, the α7 subunit was reported to be overexpressed in clear cell renal cell cancer, correlating with higher pathological grade, increased T stage, advanced TNM stage, and worse survival [72]. Additionally, the α7 subunit was associated with worse clinical features and prognosis. In tongue squamous cell cancer, its knockdown inhibited cell proliferation and stemness [73]. Similarly, in non-small-cell lung cancer, the α7 subunit promoted proliferation, apoptosis and stemness [74]. In esophageal squamous cell cancer, the α7 subunit has also served as a functional cancer stem cell surface marker [16].

#### Signaling pathways mediated by integrins α6β1, α3β1, and α7β1

It has been reported that integrin α6β1 was highly expressed in metastatic and androgen receptor-positive prostate cancer and promoted survival and resistance through PI3K and NF-κB signal pathways [14]. Multiple data demonstrate that integrin α3β1, in conjunction with CD151, governs the signaling pathways responsible for the viability of differentiating keratinocytes. Integrin α3β1 also plays a crucial function as a regulator of pro-tumorigenic pathways in skin carcinogenesis [15]. Furthermore, the α7 subunit regulates stem cell properties through the activation of the FAK-mediated signal pathways in esophageal squamous cell cancer [16].

### Integrins α10β1, α1β1, α2β1, and α11β1 in collagen-binding receptors

#### The role of integrins α10β1, α1β1, α2β1, and α11β1 in tumor progression

Collagen is as the most abundant component of the ECM, and its structure and function vary according to tissue types. Similar to other integrins, collagen-binding integrins act as bidirectional signaling receptors upon biochemical or mechanical activation [75]. Among them, integrin α10β1 is the most prevalent collagen-binding integrin in cartilage tissue, exhibiting distinct expression patterns compared to other collagen-binding integrins. Research has shown that targeting the α10 subunit with antibodies effectively inhibits adhesion, migration, proliferation and sphere formation of glioblastoma cells, providing a promising therapeutic approach for glioblastoma treatment [76, 77]. Studies have also revealed that α10 subunit expression is upregulated in malignant melanoma cells compared to primary melanocytes [78]. Integrin α10β1 promotes angiogenesis and aggregation of stromal cells, which in turn secrete tumor-promoting factors, thereby fostering ovarian tumor growth [17]. Specific inhibitors of integrin α1β1 can reduce collagen V-driven invasion and suppress ECM-driven cancer cell invasion through paclitaxel, suggesting that integrin α1β1 also contributes to the progression of colon cancer [19]. It was suggested that integrin α1β1 also contributes to colon cancer progression [79]. Notably, both collagen-binding integrin α1β1 and integrin α2β1, as well as laminin-binding integrin α3β1, are involved in regulating tumor cell proliferation, survival and EMT processes. It was shown that cell proliferation was suppressed in the presence of the α2β1 inhibitor [80]. Buddlejasaponin IV induced anoikis by inhibiting integrin α2β1-mediated cell adhesion and signaling and inhibited lung metastasis of colon cancer cells [81]. In primary ovarian cancer, integrin α2β1 serves as a prognostic and predictive marker. progression-free survival was shorter in patients with a high integrin α2β1 expression [82]. This investigation also provided evidence that integrin α2β1-collagen interaction activated pathways relevant to mitotic hepatoma carcinoma progression. After binding to collagen, integrin α2β1 was shown to activate the pro-oncogenic YAP in hepatoma cells, which correlated well with tumor progression and outcome in patients [83]. Alternagin-C is a substance that binds to integrin α2β1 and can weaken the adhesion of triple-negative breast cancer cells to collagen matrix while stimulating the expression of transfer inhibitory factor 1 [84]. It has been revealed that integrin α2β1 is involved in protecting tumor cells from aging, and reducing the expression of integrin α2β1 triggers an atypical signaling mechanism based on AKT, resulting in the process of cellular aging [85]. Integrin α2β1 inhibition attenuated prostate cancer cell proliferation by cell cycle arrest, promoted apoptosis and reduced EMT [86]. It has been hypothesized that integrin α11β1 promoted cutaneous squamous cell cancer by regulating ECM synthesis and collagen organization within a highly dynamic and interactive tumor microenvironment (TME) [87]. It has also been found that integrin α11β1 promoted tumorigenicity and metastasis in non-small cell lung cancer and controlled the stiffness of the cancer stroma [88].

#### Signaling pathways mediated by integrins α10β1, α1β1, α2β1, and α11β1

Integrin α10β1 functions as a receptor for the HU177 epitope, expressing α-smooth muscle actin in stromal cells, thereby regulating ERK-dependent migration [17]. Activation of the TRIO–RAC–RICTOR–mTOR signaling by the α10 subunit promotes tumor cell survival, and inhibitors of RAC and mTOR have shown anti-tumor effects in vivo, providing a potential therapeutic strategy for high-risk leiomyosarcoma patients [18]. Reports indicate that collagen V directly signals through integrin α1β1, driving cell migration. Additionally, collagen V increases invasion in triple-negative breast cancer cells through α1β1-mediated ERK1/2 signaling. The use of integrin α1β1 specific inhibitors suppresses paclitaxel-induced ECM-driven cancer cell invasion [19]. In colon cancer cells, another significant role of integrin α1β1 in tumorigenesis has been demonstrated through its interaction with talin and paxillin, activating FAK/Src and leading to focal adhesion clustering and activation of the p130Cas/JNK, thus promoting cancer cell invasion [20]. Research suggests that collagen I mediates osteosarcoma development through the integrin α2β1/JAK/STAT3 signaling pathway. Blockade of integrin α2β1 efficiently improved the outcome of chemotherapy and radiotherapy, which suggests new approaches for eradicating tumors in the clinic [21]. The integrin α11β1-Src-YAP1 signaling pathway is involved in resistance of melanoma to MAPK and PI3K/mTOR dual-targeted therapy [22].

## Clinical significance of integrin β1

**Fig. 2:**
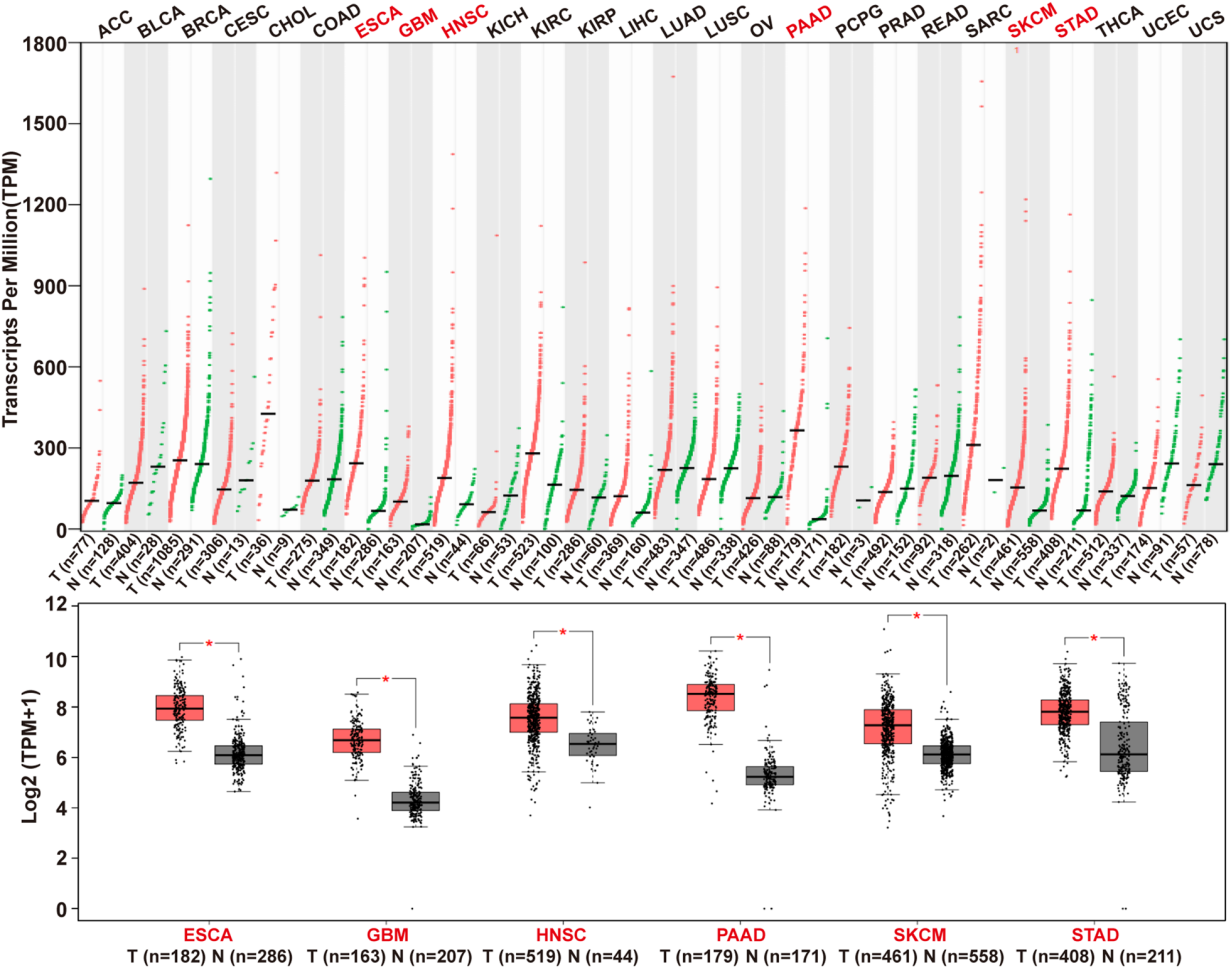
The gene expression profile across all tumor samples and paired normal tissues. Data for ITGB1 encoding integrin β1 across human cancers were collected with GEPIA. *ACC* adrenocortical cancer, *BLCA* bladder urothelial cancer, *BRCA* breast invasive cancer, *CESC* cervical squamous cell cancer and endocervical adenocarcinoma, *CHOL* cholangiocarcinoma, *COAD* colon adenocarcinoma, *ESCA* esophageal cancer, *GBM* glioblastoma multiforme, *HNSC* head and neck squamous cell cancer, *KICH* kidney chromophobe, *KIRC* kidney renal clear cell cancer, *KIRP* kidney renal papillary cell cancer, *LIHC* liver hepatocellular cancer, *LUAD* lung adenocarcinoma; *LUSC* lung squamous cell cancer, *OV* ovarian serous cystadenocarcinoma, *PAAD* pancreatic adenocarcinoma, *PCPG* pheochromocytoma and paraganglioma, *PRAD*, prostate adenocarcinoma, *READ* rectum adenocarcinoma, *SARC* sarcoma, *SKCM* skin cutaneous melanoma, *STAD* stomach adenocarcinoma, *THCA* thyroid cancer, *UCEC* uterine corpus endometrial cancer, *UCS* uterine carcinosarcoma.*, *P* < 0.05

Integrin β1 has emerged as an essential mediator in several cancers in recent years. The expression of integrin β1 in multiple cancer types is shown in Fig. 2, which indicates the applicability of integrin β1 as a therapeutic target and underlines the requirement for patient stratification in future clinical studies. For example, in esophageal cancer, high expression of integrin β1 is related to worse overall survival, and targeting integrin β1 alleviates tumor metastasis and chemotherapy resistance of patients [89, 90]. Combined inhibition of the integrin β1 and the stress-mediator JNK induces radiosensitization, which is caused by defective DNA repair associated with chromatin changes, enhanced ataxia-telangiectasia mutated phosphorylation and prolonged G2/M cell cycle arrest in glioblastoma [91]. Eke et al*.* have reported that compared with EGFR single inhibition, the combination of integrin β1 and EGFR targeting resulted in enhanced cytotoxicity and radiosensitization of head and neck cancer cells, which responded with FAK dephosphorylation [92]. In addition, the combination of gemcitabine and hERG1/integrin β1 complex antibody reduced the volume of tumor masses and produced an increase in survival without significant toxic side effects in pancreatic cancer [93]. However, in melanomas, although the combination of MAPK and PI3K/AKT inhibitors was successfully used in preclinical experiments and early clinical trials, dual-drug resistance was inevitably observed. Co-targeting MAPK/PI3K pathway with integrin β1 synergistically inhibited the proliferation of melanoma cells [22]. Moreover, stabilizing the expression of integrin β1 on the surface of gastric cancer cells led to drug resistance through activation of the FAK-YAP1 signaling pathway. This finding provides a potential avenue for gastric cancer chemotherapeutics [94].

Nevertheless, the relationship between integrin β1 and clinical characteristics of patients is controversial and the prognostic significance of increased integrin β1 expression also varies depending on the type of cancer (Table 3). It has been reported that integrin β1 exerts an influence on prognosis in periampullary cancer but not in ductal pancreatic cancer [95]. Other studies have demonstrated that integrin β1 was strongly associated with a shorter survival time of gastric cancer patients [96]. Sun et al*.* have proved that high expression of integrin β1 was linked to poorer overall survival in lung cancer [97]. Immunohistochemistry analyses have revealed that the highest integrin β1 intensity score was associated with significantly decreased 10-year overall survival and disease-free survival in invasive breast cancer [98]. In addition, univariate and multivariate analysis has indicated that lack of integrin β1 expression was associated with biochemical recurrence and time to recurrence after radical prostatectomy [99]. Lu et al*.* have shown that the low expression of the α8 subunit was associated with poor prognosis for overall survival and disease-free survival in clear cell renal cell cancer patients [100]. Moreover, studies have reported that integrin β1 overexpression in colorectal tumors was associated with poor prognosis, as well as aggressive clinicopathological features [101].

**Table 3:** The clinical impacts of integrin β1 in cancer patients

Tumor type	Expression	Clinical impacts	Reference
Pancreatic cancer	up	Periampullary carcinoma, poorer prognosis; ductal pancreatic carcinoma, unrelated	[95]
Gastric cancer	up	Associated with a shorter survival time	[96]
Lung cancer	up	Associated with worse overall survival	[97]
Breast cancer	up	Linked to decreased 10-year overall survival and disease-free survival	[98]
Prostate cancer	down	Associated with biochemical recurrence	[99]
Renal cell cancer	down	Positively related to prognosis	[100]
Colorectal cancer	up	Linked to poor prognosis; independently correlated with shortened overall survival and disease-free survival	[101]

## Integrin β1 and therapy

The above results all shed light on the importance of the integrin β1 molecule in tumor growth, metastasis and drug resistance and highlight the potential of integrin β1 in personalized cancer therapy. The potential clinical applications of integrin β1 as a target for cancer therapy have generated great interest and shown theoretical potential as novel drugs for anti-tumor therapy, and indeed multiple antagonists and agonists of the integrin β1 signaling pathway provide the rationale for clinical development. Integrin β1 has historically been a promising yet challenging target for the treatment of multiple cancers. For example, integrin α5β1 has been used as a targeting strategy in clinical trials for non-small cell lung cancer, pancreatic cancer, epithelial ovarian cancer, primary peritoneal carcinoma, renal cell carcinoma and melanoma. In addition, targeting integrin α4β1 was also effective in the treatment of acute myeloid leukemia and solid tumors. The ongoing clinical studies of integrin β1-targeting drugs currently being tested as disease therapies are summarized in Table 4.

**Table 4:** Integrin β1-targeting cancer therapies in clinical trials

Drug name	Drug type	Source	Target	Indication	Study status
Volociximab	Antibody	NCT00099970; NCT00100685; NCT00278187; NCT00369395; NCT00401570; NCT00516841	α5β1	Non-small cell lung cancer; pancreatic cancer; epithelial ovarian cancer or primary peritoneal cancer; renal cell carcinoma; melanoma	Phase II (terminated)
MINT-1526A	Antibody	NCT01139723	α5β1	Solid tumors	Phase I
PF-4605412	Antibody	NCT00915278	α5β1	Solid tumors	Phase I (terminated)
OS2966	Antibody	NCT04608812	β1	Glioma	Phase I
Pegylated recombinant human endostatin	Peptide	NCT01527864	α5β1	Non-small cell lung cancer	Phase II
Ac-PHSCN-NH2	Peptide	NCT00131651	α5β1	Renal cell cancer	Phase II (terminated)
AS-101	Small molecule	NCT00418249; NCT00788424; NCT00927212; NCT00926354; NCT01010373; NCT01555112; NCT01943630; NCT03216538	α4β1	Acute myeloid leukemia	Phase II (terminated)
GLPG-0187	Small molecule	NCT00928343; NCT01313598; NCT01580644	α5β1	Solid tumors	Phase I
7HP-349	Small molecule	NCT04508179	α4β1	Solid tumors	Phase I
BA 015 gene therapy	Gene therapy	NCT01764009	α5β1	Melanoma	Phase II (terminated)

Source of clinical trials information: ClinicalTrials.gov. All information is current as of October 2023. Trials in healthy volunteers only are excluded

## Discussion

In this review, we elucidate our understanding of the characteristics, ligands, signaling pathways and biological functions of integrin β1, which can be classified into four receptors; namely, the RGD-binding receptors, leukocyte-specific receptors, laminin-binding receptors and collagen binding receptors according to the specificity of the ligands [102]. The current investigation provides evidence that the integrin β1–ECM interaction activates FAK, MAPK, PI3K-AKT, and other pathways for tumor growth, metastasis, invasion and angiogenesis [103]. Moreover, integrin β1 also confers tumor cell chemoresistance, radioresistance, and immunoresistance [104]. Binding of integrin β1 to collagen I induces breast cancer cell insensitivity to cisplatin, doxorubicin, and mitoxantrone cytotoxicity [105]. Integrin β1 molecules promote radiotherapy resistance by repairing DNA double-strand breaks and induce pro-survival signaling through the engagement of FAK and JNK signal pathways in head and neck cancer [106, 107]. The c-Met/integrin β1 complex is formed during the metastasis and invasion of glioblastoma, liver cancer and breast cancer, and its decoupling helps to alleviate drug resistance [108]. Xu et al*.* have reported that higher expression of integrin β1 was associated with worse pathological G-staging and tumor T-staging, which was positively correlated with CD8^+^ T cells in gastric cancer [109]. Therefore, targeting integrin β1 provide therapeutic benefit to overcome multiple drug resistance. The expression of integrins varies greatly between normal and tumor tissues and is related to the type of cancer. In addition, different α subunits combining with the same β subunit may play very different roles. For instance, integrin α10β1 plays an important role in the progression of melanoma, while integrin α9β1 is strongly related to breast cancer, ovarian cancer and colon cancer [4]. Hence, it is critical for different tumor types to be considered in personalized targeted therapy. Currently, there are about 90 kinds of integrin-based therapeutic drugs or imaging agents which have been applied in clinical research, including small molecules, antibodies, synthetic mimic peptides, antibody–drug conjugates, chimeric antigen receptor T-cell therapy and imaging agents, among others [4].

## Conclusions

Considering the potential function of integrin inhibition in overcoming acquired resistance to chemotherapy, radiotherapy and immunotherapy, combination therapy of anti-tumor drugs with integrin antagonists is expected to overcome the current difficulty of drug resistance in tumors. Also, this study indicates the applicability of integrin β1 as a therapeutic target and highlights the need for patient stratification according to expression of different integrin receptors in future clinical studies.

## Data Availability

Not applicable.
